# Supplementary material for: Virus-Subtype-Specific Cellular and Humoral Immune Response to a COVID-19 mRNA Vaccine in Chronic Kidney Disease Patients and Renal Transplant Recipients
Source: Microorganisms. 2023 Jul 5;11(7):1756. doi: 10.3390/microorganisms11071756 (PMC10383116; doi:10.3390/microorganisms11071756)
Supplement: Supplementary file 1 [file microorganisms-11-01756-s001.zip › microorganisms-2411124-supplementary.pdf]

Supplementary Table S1: Patients quantitative antibodies in BAU/ml measured at each time point (t_1 = day of first vaccine dose; t_2 = approx. 3 months after vaccination).

Case	Vasculitis		CKD		KT	
	**t_1**	**t_2**	**t_1**	**t_2**	**t_1**	**t_2**
1	0	0	0	3053.7	0	72.7
2	0	0	0	4106.8	0	27.9
3	0	44.3	3.1	3955.7	0	0
4	0	816.7	0	2254.3	0	31.0
5	0	0	0	3853.4	5.8	27.8
6	0	170.7	0	3677.6	0	83.3
7	0	342.3	0	3732.8	0	22.7
8			0	3468.2	0	613.7
9			0	951.5	0	26.9
10			0	2744.3	0	181.8
11			0	4139.5	0	16.2
12			0	1440.1	0	440.3
13			0	11360	0	534.8
14			0	3800.9	0	0
15			0	654.8	0	0
16			0	3178.9	0	0
17			0	6099.4	0	16.0
18			0	1255.6		

Supplementary Table S2: *List of patients' NT50 values at each time point (t_1 = day of first vaccine dose; t_2 = approx. 3 months after first vaccine dose).*

	NT50 t_1			NT50 t_2		
	**WT**	**Delta**	**Omicron**	**WT**	**Delta**	**Omicron**
**Vasculitis**	1.0	1.0	9.2	1.0	1.0	13.6
	1.0	1.0	1.0	1.0	1.0	1.0
	1.0	1.0	1.0	5.5	1.0	1.0
	1.0	1.0	1.0	203.6	283.5	1.0
	1.0	1.0	1.0	1.0	1.0	1.0
	1.0	1.0	1.0	1.0	1.0	1.0
	1.0	1.0	1.0	93.9	75.9	1.0
**CKD**	1.0	1.0	1.0	310.1	619.8	74.0
	1.0	1.0	1.0	1152.0	768.0	32.4
	1.0	1.0	1.0	1502.1	553.4	36.1
	1.0	1.0	1.0	1531.9	446.5	41.8
	1.0	1.0	1.0	2442.7	698.7	40.6
	1.0	1.0	1.0	2121.8	302.5	8.3
	1.0	1.0	1.0	820.6	316.7	10.1
	1.0	1.0	1.0	2235.6	315.7	1.0
	1.0	1.0	9.2	512.0	178.1	9.2
	1.0	1.0	1.0	1272.1	240.5	1.0
	1.0	1.0	1.0	1187.7	402.1	1.0
	1.0	1.0	1.0	439.6	266.7	26.5
	1.0	1.0	1.0		2725.2	163.1
	1.0	1.0	1.0	5525.6	1353.5	31.6
	1.0	1.0	1.0	491.4	306.2	1.0
	1.0	1.0	9.2	2267.9	626.3	63.5
	1.0	1.0	1.0	2866.2	813.9	17.4
	1.0	1.0	1.0	635.7	373.0	18.6
**KT**	1.0	1.0	1.0	126.8	1.0	1.0
	1.0	1.0	1.0	1.0	1.0	1.0
	1.0	1.0	1.0	1.0	1.0	1.0
	1.0	1.0	1.0	357.0	1.0	1.0
	1.0	1.0	23.7	5.7	1.0	22.7
	1.0	1.0	1.0	150.9	28.0	1.0
	1.0	1.0	1.0	132.7	29.8	1.0
	1.0	1.0	1.0	197.1	641.7	1.0

	1.0	1.0	1.0	209.9	48.2	1.0
	1.0	1.0	1.0	128.0	29.1	1.0
	1.0	1.0	1.0	1.0	4.1	1.0
	1.0	1.0	5.0	212.1	139.4	1.0
	1.0	1.0	1.0	34.8	305.7	1.0
	1.0	1.0	1.0	1.0	1.0	1.0
	1.0	1.0	27.3	1.0	1.0	27.3
	1.0	1.0	1.0	1.0	1.0	1.0
	1.0	1.0	1.0	1.0	1.0	1.0

Supplementary Table S3: *Neutralisation titre according to underlying disease, taking into account the virus variant as well as the time of sampling (t1 = day of first vaccine dose; t2 = approx. 3 months after first vaccine dose)*

**Disease and virus variant$_{\text{time point}}$**	**Median NT50 [25% quartile; 75% quartile]**	**Median NT90 [25% quartile; 75% quartile]**
Vasculitis		
WT_{t1}	1.0 [1.0; 1.0]	1.0 [1.0; 1.0]
WT_{t2}	1.0 [1.0; 49.7]	1.0 [1.0; 9.1]
$Delta_{t1}$	1.0 [1.0; 1.0]	1.0 [1.0; 1.0]
$Delta_{t2}$	1.0 [1.0; 38.5]	1.0 [1.0; 5.7]
$Omicron_{t1}$	1.0 [1.0; 1.0]	1.0 [1.0; 1.0]
$Omicron_{t2}$	1.0 [1.0; 1.0]	1.0 [1.0; 1.0]
CKD		
WT_{t1}	1.0 [1.0; 1.0]	1.0 [1.0; 1.0]
WT_{t2}	1272.1 [635.7; 2235.6]	192.0 [113.6; 266.8]
$Delta_{t1}$	1.0 [1.0; 1.0]	1.0 [1.0; 1.0]
$Delta_{t2}$	424.3 [308.6; 680.6]	134.7 [78.6; 176.1]
$Omicron_{t1}$	1.0 [1.0; 1.0]	1.0 [1.0; 1.0]
$Omicron_{t2}$	22.5 [8.6; 39.5]	6.1 [1.0; 9.4]
KT		
WT_{t1}	1.0 [1.0; 1.0]	1.0 [1.0; 1.0]
WT_{t2}	34.8 [1.0; 150.9]	1.0 [1.0; 1.0]
$Delta_{t1}$	1.0 [1.0; 1.0]	1.0 [1.0; 1.0]
$Delta_{t2}$	1.0 [1.0; 29.8]	1.0 [1.0; 1.0]
$Omicron_{t1}$	1.0 [1.0; 1.0]	1.0 [1.0; 1.0]
$Omicron_{t2}$	1.0 [1.0; 1.0]	1.0 [1.0; 1.0]

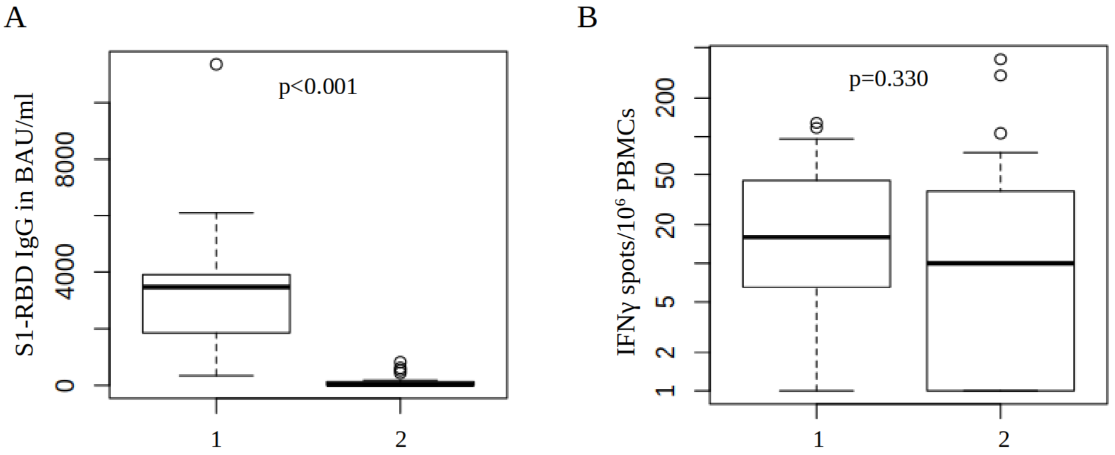

Supplementary Figure S1: *Comparison of immune responses in non-immune-suppressed (1) versus immune-suppressed (2) patients independent of the underlying cause of disease at three month of follow up: A) quantitative anti-SARS-CoV-2 antibody levels, B) T-cell response to S1 protein by IFNγ ELISpot. Boxplots are representing the median, 25-75% quartiles as well as minimum and maximum whiskers. Outliers are identified if greater/less than third quartile ± 1.5 interquartile range. Mann-Whitney-U test was used to calculate p-values.*

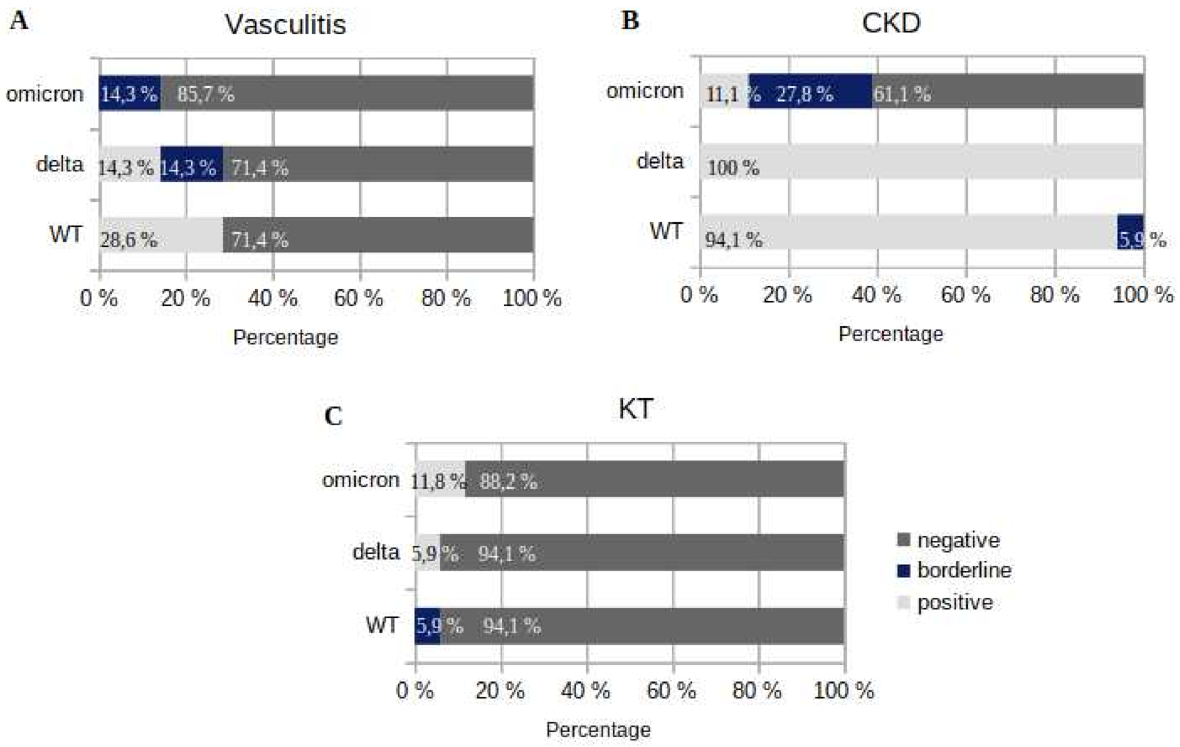

Supplementary Figure S2: 90% neutralising antibody titers (NT90): Percentages of patients with negative,

borderline and positive NT90 are shown, taking into account the respective underlying disease (A-C) and the virus variant.
